# The application of nanogenerators and piezoelectricity in osteogenesis

**DOI:** 10.1080/14686996.2019.1693880

**Published:** 2019-11-19

**Authors:** Fu-Cheng Kao, Ping-Yeh Chiu, Tsung-Ting Tsai, Zong-Hong Lin

**Affiliations:** a Institute of Biomedical Engineering, National Tsing Hua University, Hsinchu, Taiwan; b Department of Power Mechanical Engineering, National Tsing Hua University, Hsinchu, Taiwan; c Frontier Research Center on Fundamental and Applied Sciences of Matters, National Tsing Hua University, Hsinchu, Taiwan; d Department of Orthopaedic Surgery, Spine Section, Chang Gung Memorial Hospital, Taoyuan, Taiwan

**Keywords:** Osteogenesis, piezoelectricity, bone remodeling, nanogenerator, 102 Porous / Nanoporous / Nanostructured materials, 202 Dielectrics / Piezoelectrics / Insulators

## Abstract

Bone is a complex organ possessing both physicomechanical and bioelectrochemical properties. In the view of Wolff’s Law, bone can respond to mechanical loading and is subsequently reinforced in the areas of stress. Piezoelectricity is one of several mechanical responses of the bone matrix that allows osteocytes, osteoblasts, osteoclasts, and osteoprogenitors to react to changes in their environment. The present review details how osteocytes convert external mechanical stimuli into internal bioelectrical signals and the induction of intercellular cytokines from the standpoint of piezoelectricity. In addition, this review introduces piezoelectric and triboelectric materials used as self-powered electrical generators to promote osteogenic proliferation and differentiation due to their electromechanical properties, which could promote the development of promising applications in tissue engineering and bone regeneration.

## GRAPHICAL ABSTRACT

**Figure:**
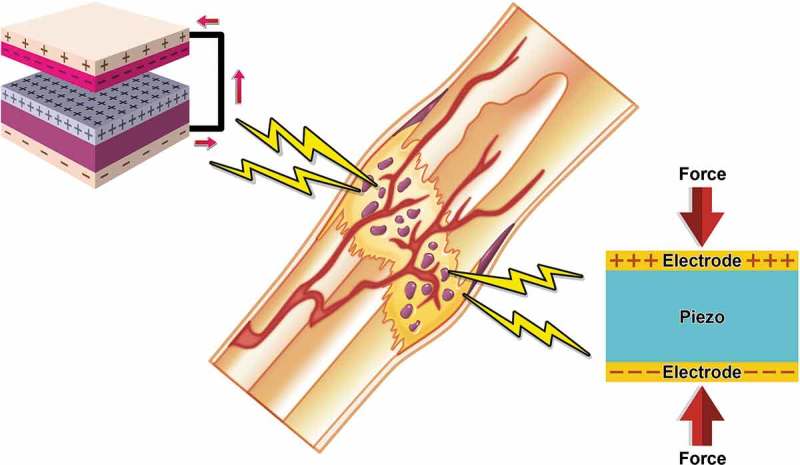

## 1. Introduction

**Figure 1.**
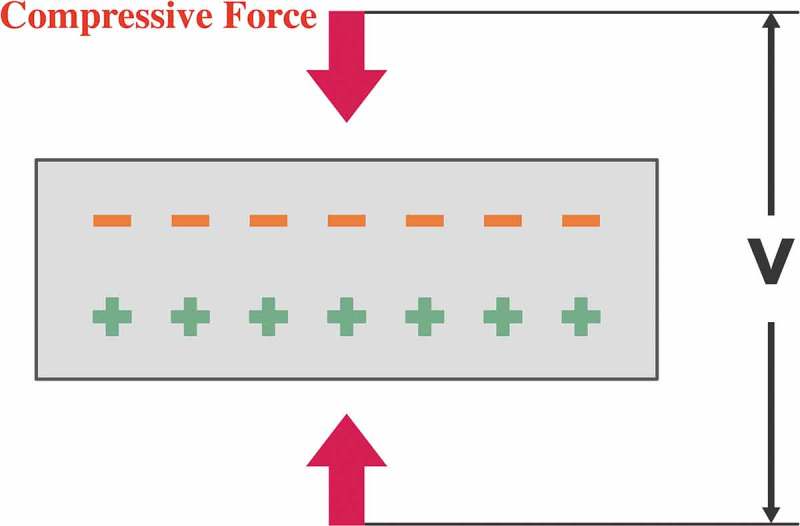
Mechanical force can push some atoms closer together or further apart in piezoelectric materials, upsetting the balance of positive and negative forces, and causing net electrical charges to appear outside.

Piezoelectricity, also referred to as the piezoelectric effect, is the ability of certain solid materials to generate an electric field in response to mechanical deformation. It is understood that this phenomenon results from the linear electromechanical interaction between the mechanical and electrical state in crystalline materials [1]. In most crystals (such as metals), the basic repeating unit is symmetrical. However, in piezoelectric crystals, it is inversely arranged, but their electrical charges are perfectly balanced and electrically neutral. The origin of the electric field in piezoelectric material is a break in the inversion symmetry, pushing some of the atoms closer together or further apart, upsetting the balance of positive and negative forces, and causing net electrical charges to appear. This effect carries through the whole structure, so net positive and negative charges appear on the opposite, outer faces of the crystal [2](Figure 1). To better describe and discuss the piezoelectric effects of different materials, we have categorized the materials into three groups according to their nature as natural, biological, and synthetic materials. Quartz, berlinite, sucrose and topaz are examples of naturally occurring materials. Collagen of bone, silk, tooth dentin and enamel belong to biological ones. Examples of synthetic materials include barium titanate and lead zirconate titanate [3].

Bone is a rigid organ that supports and protects various parts of the body. It is highly hierarchical in structure and composed of an extracellular matrix and cellular components: osteoblasts, osteoclasts, osteocytes and bone marrow cells (including hematopoietic cells). The extracellular matrix consists of 65% mineral matrix and 35% organic matrix [4]. Type I collagen makes up about 90% of the organic matrix and possesses a triple helical structure that contributes tensile strength to the extracellular matrix. Inorganic minerals, which are responsible for the compressive strength of bone, are incorporated with the collagen fibrils in the form of calcium hydroxyapatite [5]. Osteoblasts arise from mesenchymal stem cells and are responsible for bone formation. On the other hand, osteoclasts are multinucleated cells deriving from hematopoietic progenitors in the bone marrow and are responsible for bone resorption. Osteocytes are thought to be mechanosensor cells that control the activity of osteoblasts and osteoclasts. They are embedded in lacunae with long processes located in small channels called canaliculi. Canaliculi are considered the lifelines that permit nutrients, oxygen, and waste products to be exchanged with the blood vessels within the Haversian canal, Volkmann canal, and osteocytes. When a bone is loaded, the interstitial fluid within the lacuna and canaliculi is squeezed through a thin layer of non-mineralized matrix surrounding the cell bodies and cell processes toward the Haversian or Volkmann channels. This flow of fluid mobilizes the cell surface glycocalyx and initiates biochemical processes promoting osteogenesis [6].

According to Wolff’s Law, bone can respond to mechanical loading and is subsequently reinforced in the areas of stress [7]. In the view of a biophysical concept, bone remodeling is the interaction between osteoblasts and osteoclasts, which serves to regulate the process of bone formation and resorption. Fracture healing is a proliferative physiological process in which osteoblasts are activated to facilitate the repair of the fracture site. However, it remains undetermined exactly how a bone is capable of responding to mechanical signals and specifically how osteoclasts and osteoblasts can perceive such forces. Importantly, bone was first determined to be a piezoelectric material in the 1960s [8], with demonstrable electrical polarization when it is mechanically deformed. The occurrence of piezoelectricity, therefore, is one theory that could potentially explain how electrical signals and mechanical loads are involved in the adaptation of bone.

## 2. Piezoelectricity in bone tissue

The piezoelectric effect of bone was first discovered in 1957 [8]. Eiichi Fukada measured the piezoelectric constants of bone by way of three different experiments: measurements of the static direct effect, the dynamic direct effect, and the dynamic converse effect. The piezoelectric effect appeared when the shearing force was applied to the collagen fibers to make them slip past each other. The origin of piezoelectricity in bone can be ascribed to the piezoelectric effect of the crystalline micelle of the collagen molecules. Collagen fibers, a major organic matrix component with a triple helical structure, is highly oriented and patterned in bone. This construct could provide a collective, cohesive response to mechanical stresses, such as tension, compression, or torsion. The relationship between electrical polarization and such stresses likely results from the sliding of the collagen fibers against each other. The direct and converse piezoelectric effects of collagen were first observed in the Achilles tendon of ox and horse according to the study by Eiichi Fukada in 1964 [9]. The polarization or displacement of hydrogen bonds in the polypeptide chains of the collagen crystals can result in a piezoelectric effect. Deposition of hydroxyapatite stimulated by collagen piezoelectricity in deformed cortical bone was first investigated in the study by Noris-Suarez et al. in 2007 [10]. They used scanning electron microscopy (SEM), thermally stimulated depolarization current (TSDC), and differential scanning calorimetry (DSC) to evaluate the mineralization process induced as a consequence of the piezoelectricity effect. Mineralization occurred predominantly over the compressed side of the bone collagen due to the effect of collagen piezoelectricity, as was demonstrated via SEM. The DSC and TSDC results revealed a reduction in the collagen glass transition as the mineralization process advanced. The authors concluded that the piezoelectric dipoles produced by deformed collagen could induce the precipitation of hydroxyapatite by electrochemical means, even without osteoblasts present. Furthermore, calcium hydroxyapatite plays an important facilitating role in collagen piezoelectricity. There are two major effects of calcium hydroxyapatite that contribute to collagen piezoelectricity. One is that the mineral crystal structure of calcium hydroxyapatite displays a high elastic modulus compared to other biological molecules and bone [11]. This allows the collagen fibers to respond mechanically to loads onto the bone locally and bear the greatest strain of all the molecules within the solid matrix, thus generating the needed deformation required for a piezoelectric effect. The other is water resistance and restriction of hydroxyapatite to collagen. A number of physical observations support the dehydrating effect of calcium hydroxyapatite, including the result that collagen in calcified bone does not shrink and there exists a higher rate of water resorption in decalcified bone than that measured in calcified bone [12].

## 3. Effects of piezoelectricity on osteocytes

The piezoelectric effect introduced by mechanical force toward the collagen inside of bone has been shown to have a strong effect on the activation of osteocytes. The re-organization of a dipole moment is triggered by the compressive force on collagen, thus generating negative charges on the surface [13]. The negative charges can open the voltage-gated calcium channels on osteocytes. After the opening of voltage-gated calcium channels, cascades of signaling pathways are triggered. The active channels promote the influx of extracellular calcium and further activate calmodulin, which subsequently stimulates the activation of calcineurin. This could ultimately lead to the activation of Ras and the extracellular signal-related protein kinase (ERK) signaling pathway that is critical for Runx2 activation and induction of several growth factors, including transforming growth factor β and bone morphogenetic protein [14,15]. Growth factors can promote osteoblast activation, proliferation, differentiation, extracellular matrix deposition, and subsequent bone formation. However, the deformation of bone tissue during normal locomotion does not exceed 0.1%, and *in vitro* studies have shown that at least 1 to 10% deformation of bone tissue is necessary for osteocytes to respond to a mechanical strain [16,17]. Mechanical strains resulting in such deformations would cause the bone to fracture. This obvious paradox between the mechanical strains on the macroscopic and microscopic levels is well justified and has been investigated via an experimental mathematical model developed by You et al., in which it is discovered that osteocytes would amplify the mechanical strains generated by physical activity in the canalicular system due to fluid drag on the pericellular matrix [17].

## 4. Bone fracture healing

Bone fracture healing is a physiologic process that replaces the injured bone with new bone, thereby renewing the biologic and mechanical properties to a pre-injured state. These events occur in a sequential progression of overlapping processes and require the coordinated contributions of a variety of cellular activities. Bone tissue damage initiates a series of events, including hemorrhage, coagulation, inflammation, angiogenesis, bone repair, and progressive remodeling of the new bone. Mechanical stability of fracture sites decides the manner of bone healing. In rigidly stable mechanical environments, bone has the potential to heal via intramembranous ossification without callus formation. In 1949, rigid fixation without callus formation was first described as ‘primary bone healing.’ Primary bone healing involves a direct attempt by the cortex to reestablish itself in circumstances of strains less than 5% with compressive or tensile pressures of 0.15 MPa or less [18]. Primary bone healing is driven by remodeling osteoclasts and osteoblasts bridging the fracture gap and rejoining the fractured fragments [19]. This process occurs exclusively when there are simple fracture patterns and anatomic restoration of the fracture fragments with rigid internal fixation [18]. Where there is close contact between the fractured bone ends, lamellar bone can form directly across the fracture line by extension of osteons. Without grossly visible callus formation, osteoclasts cut across the fracture line, and osteoblasts then follow the osteoclasts to deposit new bone, and this occurs along with angiogenesis. If the fracture gaps prevent direct extension of osteons across the fracture site, osteoblasts fill the defects with woven bone. New lamellar bone is thus formed, and the fracture is bridged. Haversian remodeling starts reestablishing a normal cortical bone structure after the gaps are filled with woven bone.

**Figure 2.**
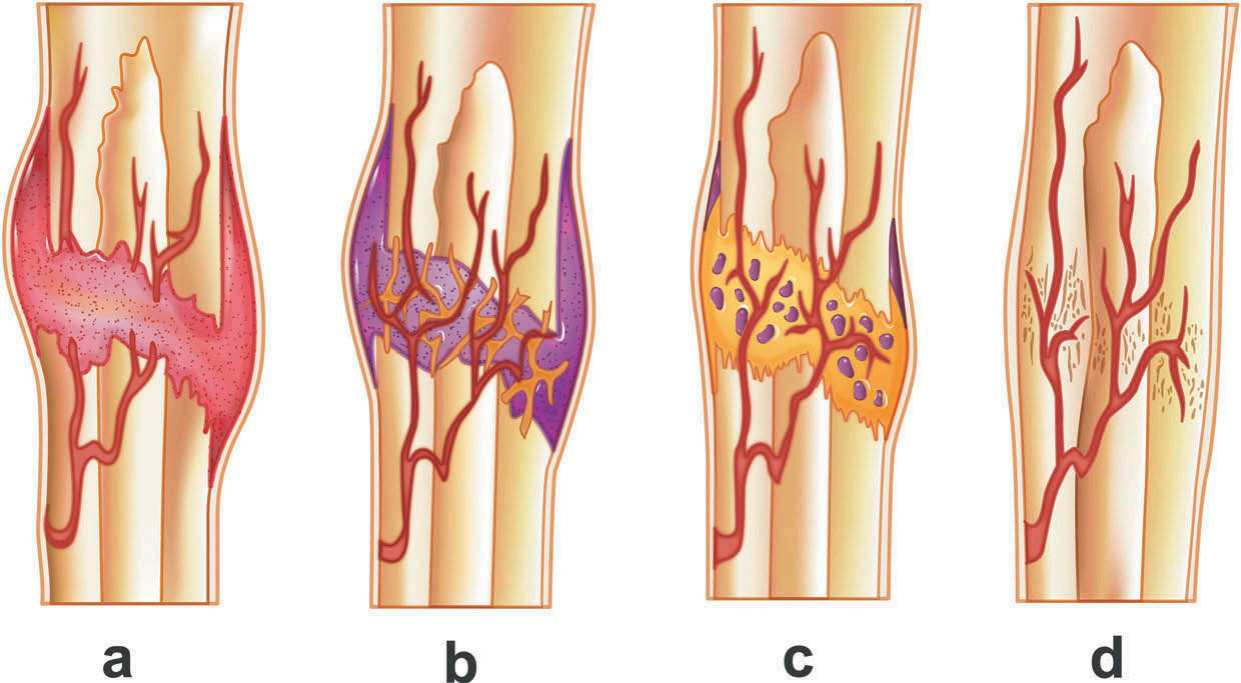
Four major phases of secondary bone healing: (a) inflammatory phase, (b) early callus phase, (c) mature callus phase, and (d) remodeling phase.

However, some fracture healings can not be treated with rigidly stable managements, as most fractures need to be treated with bracing that involves some degree of motion, including cast immobilization, intramedullary nails, bridge plating, and external fixation devices. Therefore, primary bone healing is rare, and the majority of fracture healing proceeds via secondary bone healing, or endochondral ossification, which occurs via a cartilage callus. There are four major phases of secondary bone healing, which include the inflammatory phase, early callus phase, mature callus phase, and remodeling phase. The inflammatory phase is characterized by an acute bone marrow response, post-damaged inflammation, and hematoma formation immediately following the fracture and up to 3–4 days after (Figure 2(a)). The damaged tissue releases proinflammatory mediators, such as interleukin 1 (IL-1), IL-6, and tumor necrosis factor alpha (TNF-α), to initiate the repair process [20]. The second stage is the early callus phase. This phase is predominated by soft cartilage callus formation, angiogenesis, and chondrogenesis at the fracture gap [21] (Figure 2(b)). Subsequently, the cartilaginous matrix is mineralized to begin the third phase, the mature callus phase. At this point, the chondrocytes undergo apoptosis and osteoblasts infiltrate the callus. The primary bone is laid down on these surfaces [22] (Figure 2(c)). In the last phase or remodeling phase, the newly formed woven bone is progressively replaced by mature lamellar bone, ultimately restoring the original cortical structure [23] (Figure 2(d)).

## 5. Piezoelectric effects in bone remodeling

**Figure 3.**
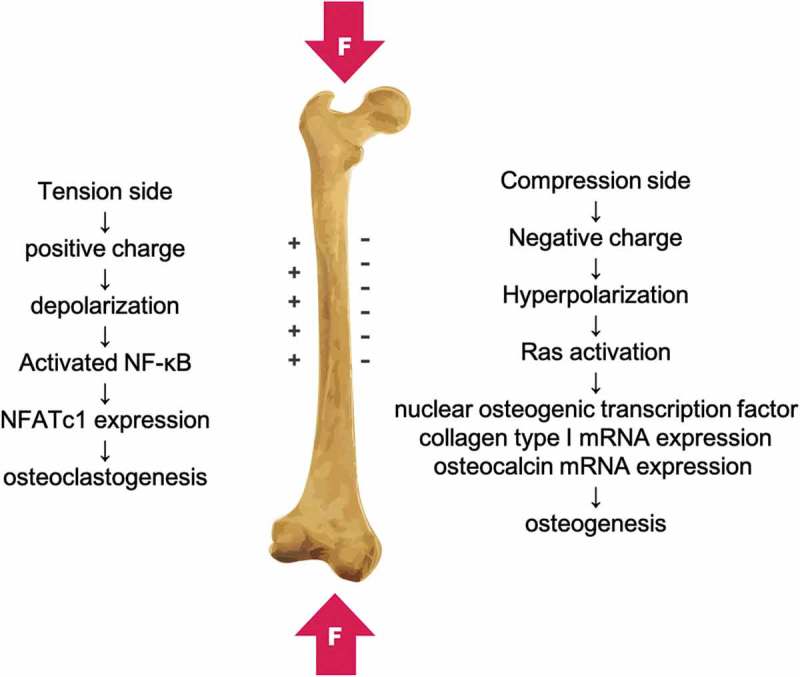
The piezoelectricity induced by mechanical deformation of bone is a negative electrical charge in areas of bone compression and a positive charge in the areas of traction.

Bone, as a mechanosensitive organ, reacts to mechanical strain via a series of cytokines and signaling crosstalk between them. Bone remodeling happens after fracture healing and also in each moment whenever bone is dynamically compressed during normal activity, as it is a lifelong process to keep bone tissue at homeostasis. There are two different mechanisms for bone remodeling: the cellular response of osteoblasts and osteoclasts to several key cytokines, and the electrochemical process due to the generation of piezoelectric dipoles. These processes are usually coupled because the piezoelectric potential produced by the deformation of bone tissue can influence the activity of osteocytes inside the bone matrix. The osteocytes can sense the mechanical force via their processes in the canaliculi and then produce several cytokines to regulate osteoclast-mediated bone resorption and osteoblast-mediated bone formation [6,24]. The piezoelectricity induced by mechanical deformation of bone generates a negative electrical charge in areas of bone compression and a positive charge in the areas of traction [25] (Figure 3). The ion channels of the osteocytes can be activated in response to both mechanical stimuli and piezoelectric currents, resulting in hyperpolarization (in the area of a negative charge) or depolarization (in the area of a positive charge) of the plasma membrane [26]. Hyperpolarization of the cell membrane potential promotes osteogenesis and osteogenic differentiation of bone marrow stem cells due to Ras activation, resulting in induction of nuclear osteogenic transcription factor, collagen type I mRNA expression, osteocalcin mRNA expression, and terminal bone matrix deposition [27,28]. RANK (receptor activator of nuclear factor kappa B) is known to play a critical role in osteoclastogenesis [29], and membrane depolarization and Ca^2+^ influx can lead to the activation and expression of nuclear factor kappa B (NF-κB) [30]. Activated NF-κB stimulates the key osteoclastogenesis regulator, the nuclear factor of activated T-cells cytoplasmic 1 (NFATc1). Subsequently, NFATc1 induces numerous osteoclast-specific target genes that are responsible for cell fusion and function after it translocates into the nucleus [31].

## 6. The application of nanogenerators for osteogenesis

**Figure 4.**
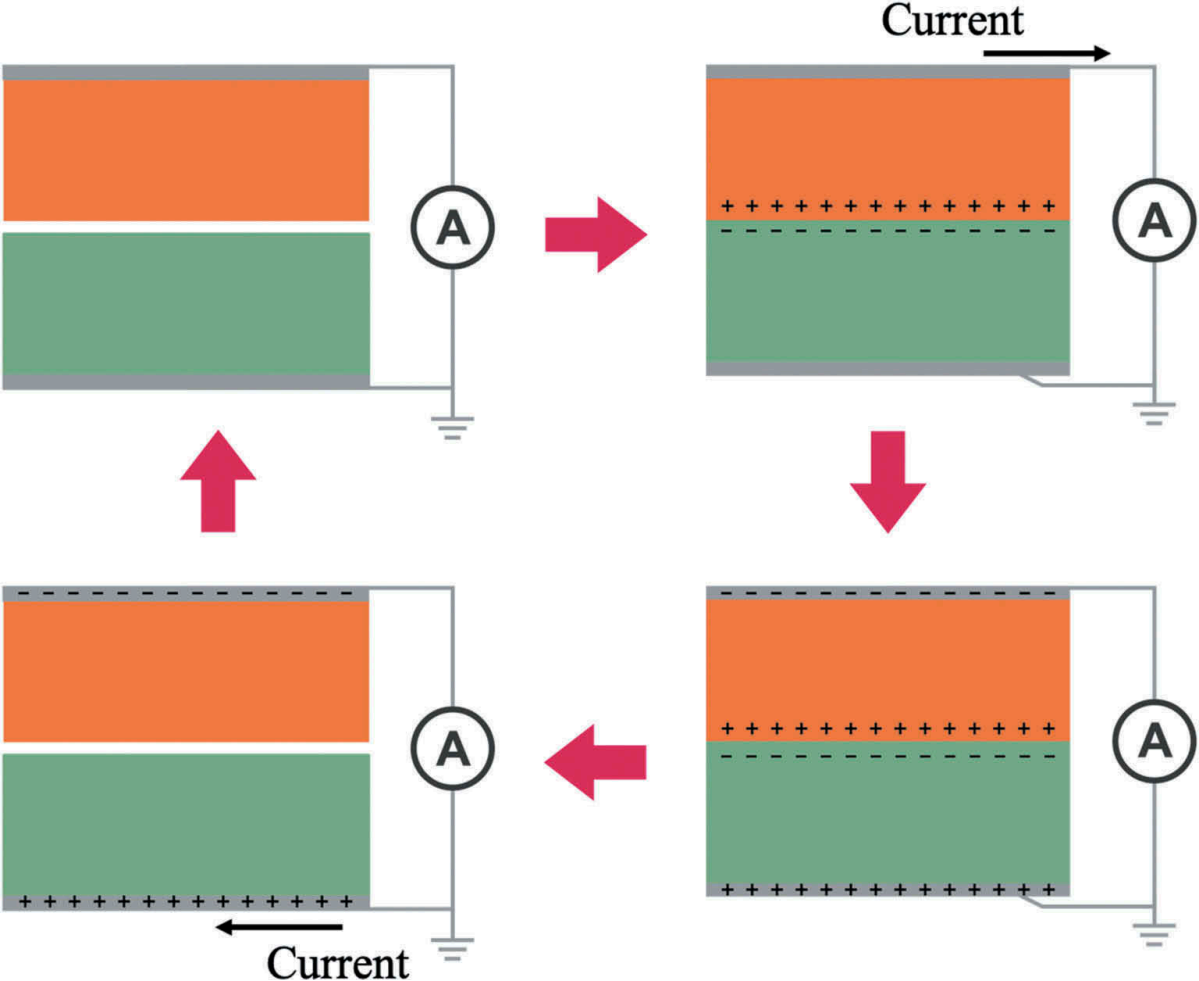
A potential is created by the triboelectric effect due to charge transfer between two dissimilar materials. The contact-induced triboelectric charges can introduce a potential drop when the two surfaces are separated by mechanical force, generating electrical currents between two electrodes set on the surfaces of the two materials.

In recent years, numerous studies have focused on the development of nanogenerators for self-powered systems. Many of these have been applied in biomedical fields and tissue engineering with great success [32–37]. In the orthopedic field, osteogenesis has been shown to be stimulated with negative electrical charges with currents between 5 and 100 μA [38]. According to the different approaches used to convert ambient mechanical energy into electricity, there are two types of nanogenerators, the triboelectric nanogenerator (TENG) and the piezoelectric nanogenerator (PENG). The coupling of contact electrification and electrostatic induction is the key concept of the TENG. The contact of two dissimilar materials causes the electrons to be transferred from one material to another due to their different capacities for attracting electrons. The contact-induced triboelectric charges can introduce a potential drop when two surfaces are separated by a mechanical force, and this will generate electrical currents between two electrodes set on the surfaces of the two materials. Therefore, the mechanical energy used to separate and contact two dissimilar materials will transform into electrical currents [39–41] (Figure 4). The working principle of the PENG involves the piezoelectric effect of piezoelectric materials under the action of a stress event. Piezoelectric materials can be combined with flexible substrates and connected electrodes to form a self-powering PENG [42]. The piezoelectric potential can then be changed to produce a current pulse when applying a dynamic external force [43].

**Figure 5.**
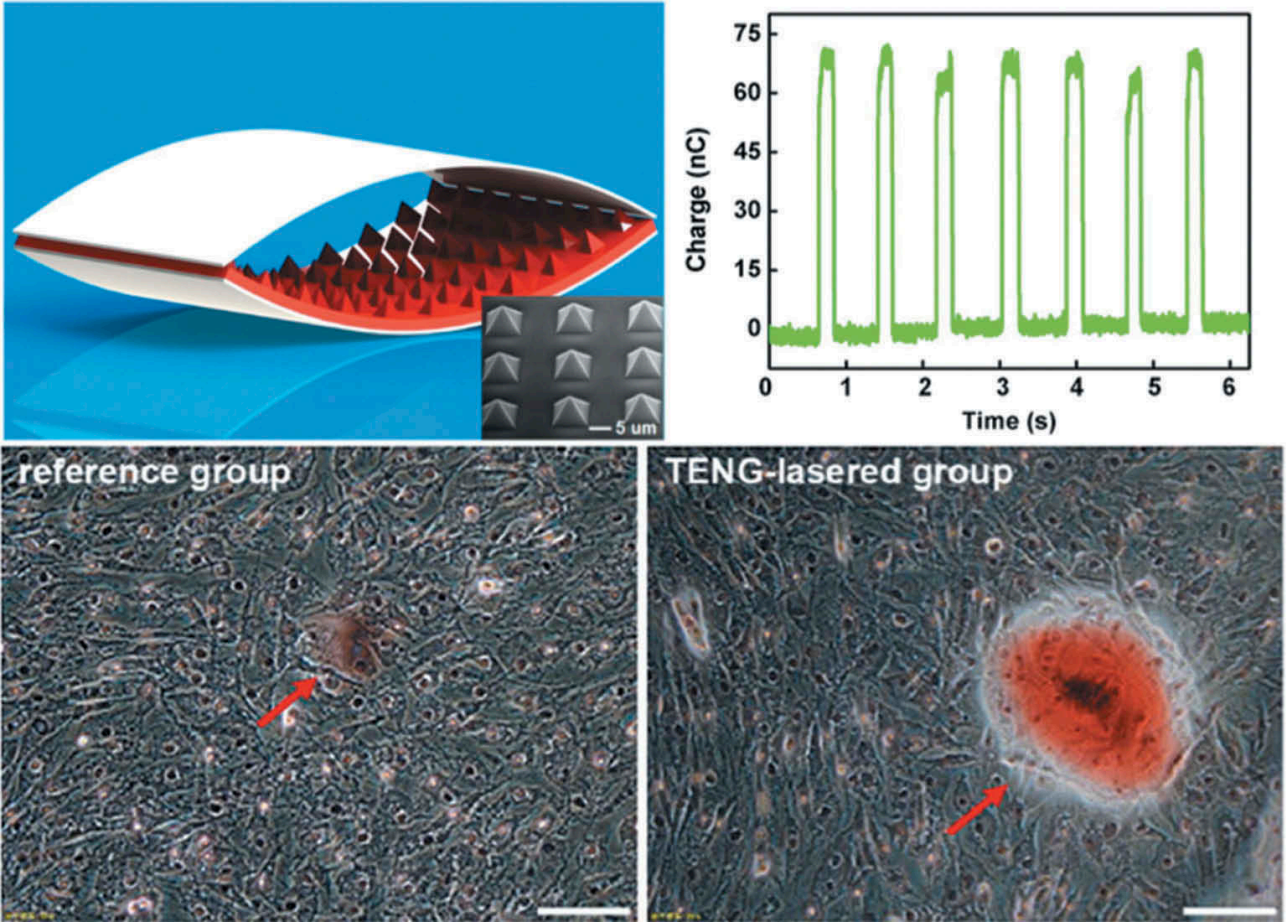
The TENG contains pyramid array patterned PDMS and indiumtin oxide (ITO) films as the friction materials and can increase the differentiation of preosteoblasts. Reproduced with permission [34]. Copyright, 2015, American Chemical Society.

A self-powered low-level laser curing system based on a flexible TENG was developed by Wei Tang et al. in 2015 to promote osteogenesis [34] (Table 1). This system can also work under the drive of a living creature’s motions. Pyramid array patterned polydimethylsiloxane (PDMS) and indiumtin oxide (ITO) films were utilized as the friction materials of the TENG (Figure 5), and testing results showed the TENG could output an I_SC_ of about 30 µA and a V_OC_ of 115 V, with the transferred charge per cycle being about 70 nC. In the biological experiments, murine calvarial preosteoblasts (MC3T3-E1) were allocated into three groups: the reference group without laser treatment, laser-irradiated group driven by TENG (TENG-lasered group, 100 pulses/day), and laser-irradiated group using a battery (battery-lasered group, 1 min/day). The cell proliferation increased by about 15% in the TENG-lasered group compared with the reference group, and the alkaline phosphatase (ALP) level of the TENG-lasered group was 16.9% higher than that of reference group after 5 days of irradiation. In addition, both of the infrared irradiation groups showed obviously larger mineral deposition than the reference group (Figure 5). The biological results revealed that this self-powered low-level laser cure system significantly accelerated the proliferation and differentiation of mouse embryonic osteoblasts. This work demonstrated great progress for the application of TENG in portable or implantable medical devices and also for their application in clinical therapy for promoting bone remodeling and osteogenesis.

**Table 1.** The application of nanogenerators in osteogenesis.

	Year	Material	Cell type	Key findings
TENG	2015 [34]	PDMS and ITO films	murine calvarial preosteoblasts	TENG-lasered group had better ALP activity and calcium deposition.
	2019 [37]	Al and PTFE films	murine calvarial preosteoblasts	The self-powered electrical stimulator promoted cell attachment, proliferation, differentiation and up-regulated the level of intracellular Ca^2+.^
PENG	2019 [36]	Zinc oxide (ZnO)	human osteoblasts	Human osteoblasts had the best cell proliferation on ZnO nanowires grown on titania nanotubes.

TENG: triboelectric nanogenerator; PENG: piezoelectric nanogenerator; ITO: indiumtin oxide; Al: Aluminum; PTFE: Polytetrafluoroethylene; ZnO: zinc oxide; PDMS: polydimethylsiloxane; ALP: alkaline phosphatase

Jingjing Tian, et al. proposed a self-powered electrical system consisting of a triboelectric nanogenerator (TENG) and a flexible interdigitated electrode for osteogenesis in vitro [37]. Aluminum film served as both the friction layer and electrode layer of the fabricated TENG, and the other friction layer of TENG was nanostructured Polytetrafluoroethylene (PTFE) film. The output voltage, current and transferred charge without rectified of the TENG were about 100V, 1.5 μA, and 21 nC respectively, which could light forty green LEDs simultaneously. MC3T3-E1 cells (murine calvarial preosteoblasts) after stimulated for 1, 3 and 6 hours, had higher percentage than control group in cell attachment, proliferation, differentiation and the level of intracellular Ca^2+^. The authors demonstrated that this self-powered electrical stimulator significantly promoted osteogenesis and had great potential for clinical therapy of osteoporosis and osteoporosis-related fractures.

**Figure 6.**
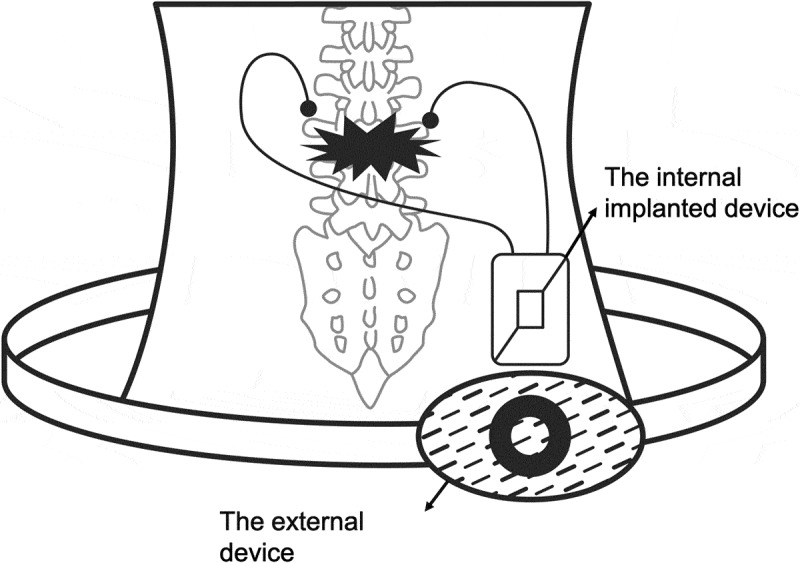
The system is composed of both implanted and external devices [36].

Webster and Kumarakuru proposed a semi-invasive system based on an implanted PENG device that could provide a direct current (DC) electrical signal inside the body to promote bone growth and fracture healing [36] (Table 1). The system was composed of both implanted and external devices (Figure 6). In the internally implanted device, piezoelectric nanowire was embedded between bottom substrates and a top protective layer. The implanted PENG device could function as a self-powered nanogenerator to sense the mechanical force applied from outside the body or via stimuli from the external device. The electrical signal generated from the PENG was conducted through electrical leads to electrodes located at a site of the intended bone growth (Figure 6). Zinc oxide (ZnO) is the preferred piezoelectric material for this nanogenerator device due to its unique semiconducting, piezoelectric property and excellent biocompatibility. In their cell proliferation study utilizing human osteoblasts, the results showed that the ZnO nanowire grown on a titanium nanotube promoted the best cell proliferation.

## 7. Piezoelectric materials for bone repair and regeneration

Piezoelectric materials have many significant applications in tissue engineering as electroactive scaffolds for use in tissue repair and regeneration. Such materials can transform mechanical strain to variable electrical stimulus, and the electrical stimulation resulting from a piezoelectric scaffold has the ability to regenerate and repair tissues via defined pathways [44]. Such piezoelectric scaffolds with optimized properties could produce suitable bioelectrical signals, similar to that of the natural extracellular matrix (ECM) observed during the processes of osteogenesis and chondrogenesis [45]. Piezoelectric materials used for bone tissue engineering are classified as piezoelectric polymers or ceramics (Tables 2 and 3), and they can be used either alone or as composites.

**Table 2.** Summary of key findings in studies involving the use of piezoelectric polymers for bone regeneration and their piezoelectric coefficients.

Material	year	Cell type or animals	Key findings	Piezoelectric coefficient (pC/N)
PVDF	2015 [50]	Human adipose stem cells (hASCs)	Increased ALP activity and osteogenic differentiation under dynamic conditions.	−32
	2017 [51]	Wistar rats	More bone defect closure and bone remodeling in femurs.	
	2012 [52]	Sheep (merino ewe)	Total bone area, new bone area, bone deposition rate, and osteopontin were higher around the piezoelectric actuator.	
PVDF-TrFE	2017 [57]	Human mesenchymal stem cell	Piezoelectric scaffolds promoted chondrogenesis at a low voltage output and osteogenesis at a high voltage.	−38
	2016 [58]	Bone marrow mesenchymal stem cells and Wistar rats	PVDF-TrFE based composite membranes produced a surface potential that enhanced osteogenesis *in vitro* and bone regeneration *in vivo*.	
PHBV	2012 [62]	SaOS-2 cells	PHBV-based scaffold promoted cell proliferation and ALP activity in osteoblastic cells.	1.3
	2013 [63]	MG-63 cells	β-Ca2SiO4/PHBV composite scaffolds upregulated the gene transcription of TGF-β1, BMP-7, and ALP to enhance cell proliferation.	
PLLA	1996 [67]	Cats, *in vivo* analysis	Fracture healing and callus formation of cat tibiae were promoted as the draw ratio of the PLLA rod increased.	−10

Osteosarcoma cell lines: SaOS-2, MG-63; *TGF-β1*: transforming growth factor-β1; BMP-7: bone morphogenetic protein-7; ALP: alkaline phosphatase

**Table 3.** Summary of key findings in studies involving the use of piezoceramics for bone regeneration and their piezoelectric coefficients.

Material	Year	Cell type or animals	Key findings	Piezoelectric coefficient (pC/N)
Barium titanate	2018 [80]	MG-63 cells	Increased cell adhesion, proliferation, and migration into barium titanate scaffolds.	191
	2016 [81]	MG-63 cells	Higher cell density, ALP, and BGP activities on porous barium titanate composites compared to dense titanate composites.	
HA (hydroxyapatite)	2007 [87]	MSCs from rabbits and U2OS cells	20 nm HA nanoparticles had the greatest potential for stimulating bone regeneration (MSCs) but inhibited the growth of osteosarcoma cells.	1.5–2.4
	2010 [89]	MC3T3-E1 osteoblast-like cells	Cell attachment, proliferation, and metabolic activities were significantly increased on the charged HA surface.	
Boron nitride	2016 [93]	Umbilical cord mesenchymal stem cells (UC-MSCs)	BNNT composite scaffolds promoted osteogenic differentiation of UC-MSCs with more calcium deposition.	0.3
	2016 [94]	MSCs from rats	MSCs had better proliferation, ALP, and osteocalcin activities on boron nitride nanotube layers.	
ZnO (zinc oxide)	2017 [100]	SaOS2 cells	Electromechanical reactions between living cells and ZnO nanosheets could stimulate the opening of calcium channels, thus enhancing cell viability, proliferation, and differentiation.	12.4
	2014 [101]	MG-63 cells	Fracture toughness, compressive strength, cell attachment, and proliferation improved when the content of ZnO increased from 0 to 2.5 wt%.	

Osteosarcoma cell lines: U2OS, MG-63, SaOS2; MSCs: mesenchymal stem cells; ALP: alkaline phosphatase; BGP: bone gla protein; BNNT: Boron nitride nanotubes

### 7.1. Piezoelectric polymers

Piezoelectric polymers are typically fabricated in three different morphologies; films, rods, or tubes/fibers [46]. Mechanically, such polymers demonstrate a high strength and high impact resistance when compared to inorganic materials. Piezoelectric polymers, such as PVDF (poly[vinylidene fluoride]), PVDF-TrFE (poly[vinylidene fluoride-trifluroethylene]), and PLLA (poly-L-lactic acid), are of great interest due to their osteogenic capacity [47]. The following are common piezoelectric polymers used in tissue engineering for bone (Table 2).

#### 7.1.1. PVDF (poly[vinylidene fluoride])

**Figure 7.**
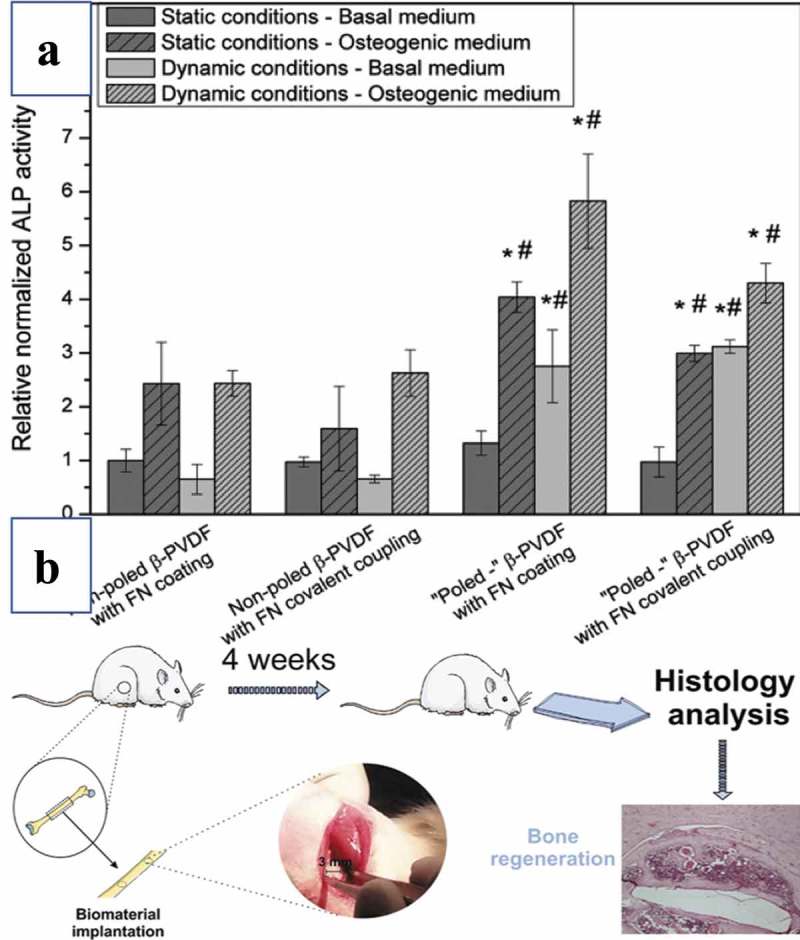
(a) The cell adhesion on ‘poled’ β-PVDF samples is stronger than in cells cultured on the other types of PVDF films [48]. Copyright, 2017, Elsevier B.V. (b) Poled and non-poled β-PVDF films have been implanted in bone defects to test their osteogenic properties in rats by assessing new bone formation [51]. Copyright, 2014, Wiley Periodicals.

PVDF is a well-known biocompatible thermoplastic that is not biodegradable and demonstrates high chemical and physical resistance [48]. Its piezoelectric coefficient (d_33_) is −32 pC/N [49], and PVDF has been used widely in the fields of biomedicine, tissue engineering, and implantable self-powered devices due to its high flexibility and lack of cell toxicity [47]. Martins et al. demonstrated the potential application of piezoelectric PVDF scaffolds for providing the necessary electromechanical stimuli for the differentiation of human adipose stem cells *in vitro* [50] (Figure 7(a)). In the *in vivo* study published by Ribeiro et al., piezoelectric PVDF films and fibers were shown to serve as suitable bone substitutes for osteogenesis. The authors used poled and non-poled β-PVDF films and randomly oriented electrospun fiber mats to test their osteogenic properties in Wistar rats by evaluating new bone formation. After 4 weeks, defects implanted with poled β-PVDF films demonstrated significantly more defect closure and bone remodeling [51] (Figure 7(b)). Both piezoelectric PVDF actuator and converse piezoelectric effects have also been used to effectively stimulate bone growth *in vivo*, as presented by Joana Reis et al [52]. In this study, PVDF actuators were implanted into osteotomy cuts in sheep femurs and tibias to test the converse piezoelectric effect to stimulate bone mechanically. After a one-month implantation, the total bone area and new bone area were significantly higher around the actuators when compared to the static controls; the bone deposition rate was also significantly higher in the mechanically stimulated areas. Based on these studies, both piezoelectric and converse piezoelectric effects appear to be effective for stimulation of bone growth.

#### 7.1.2. PVDF-TrFE (poly[vinylidene fluoride-trifluroethylene])

**Figure 8.**
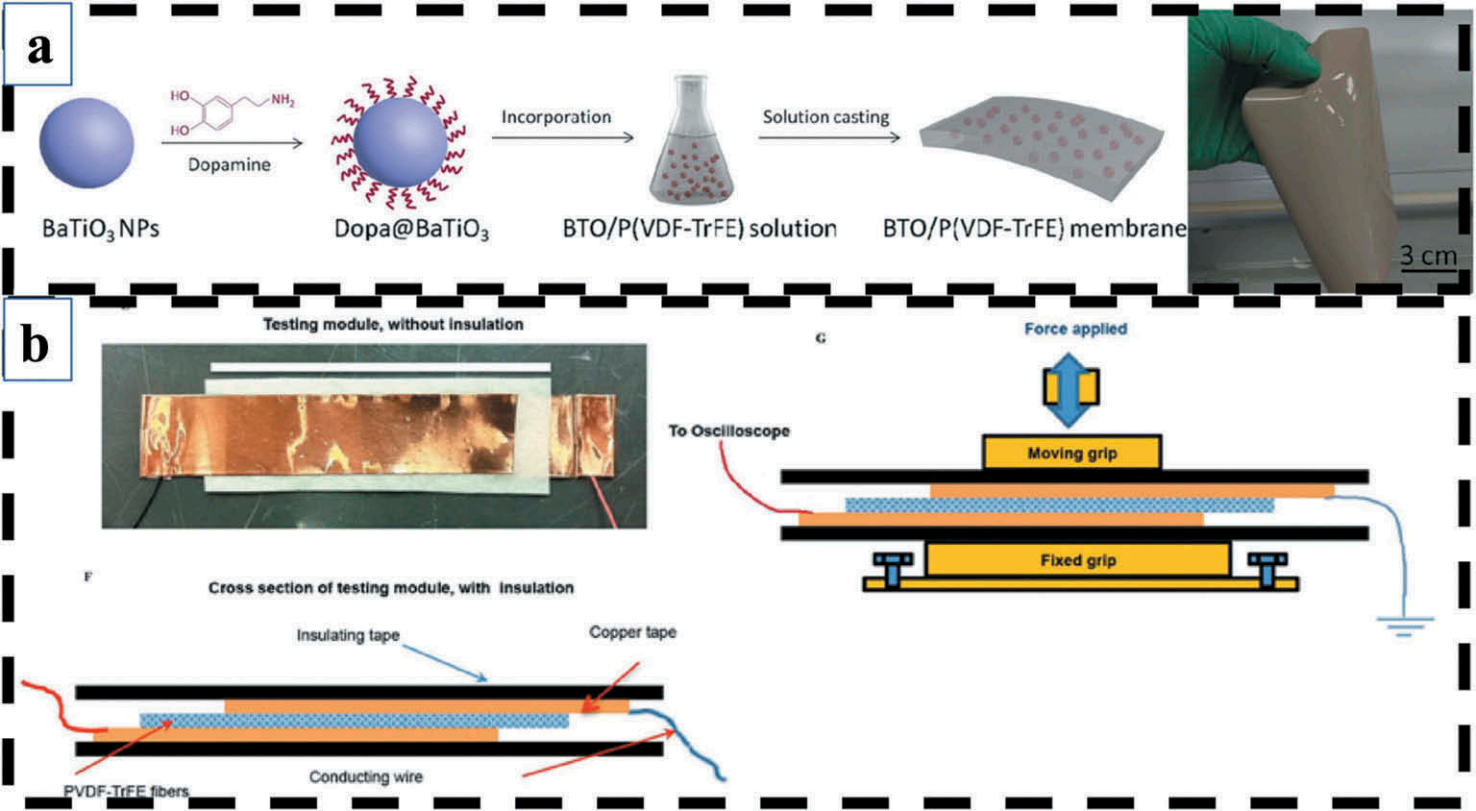
(a) PVDF-TrFE–based composite membranes have the ability to promote bone regeneration both *in vitro* and *in vivo* [58]. Copyright, 2016, American Chemical Society. (b) Piezoelectric PVDF-TrFE fibrous scaffolds can be dynamically stimulated by external forces to guide human mesenchymal stem cell differentiation [57]. Copyright, 2017, Elsevier.

PVDF-TrFE is a special polymer that automatically forms in an all-trans conformation (β-phase) and possesses a high electromechanical and piezoelectric coefficient (d_33_, −38 pC/N) at specific monomer concentrations [53–55]. This polymer has demonstrated positive effects in regard to the tissue regeneration of bone, skin, cartilage, and tendons [55]. The piezoelectric PVDF-TrFE polymer is cytocompatible in cell adhesion and proliferation [56]. According to a study published in 2017, PVDF-TrFE–based piezoelectric materials can be dynamically stimulated to guide human mesenchymal stem cell differentiation corresponding to different extracellular matrix formation. These piezoelectric scaffolds promote osteogenic differentiation when exhibiting high voltage output and promote chondrogenic differentiation at a low voltage output [57] (Figure 8(b)). In addition, PVDF-TrFE–based composite membranes have been shown to have the ability to promote bone regeneration both *in vitro* and *in vivo*. Xuehui Zhang et al. demonstrated that these membranes encourage osteogenic differentiation from bone marrow mesenchymal stem cells *in vitro* and enhance the healing of bone defect in rats [58] (Figure 8(a)).

#### 7.1.3. PHBV (poly-3-hydroxybutyrate-3-hydroxy valerate)

PHBV, a member of polyhydroxyalkanoates (PHAs), is considered a promising biopolymer due to its biocompatibility, biodegradability, and thermoplasticity [59]. Moreover, it has a high degree of crystallinity and water insolubility and demonstrates a longer degradation time than other biocompatible polymers. Its piezoelectric coefficient (d_14_, 1.3 pC/N) is similar to that of human bone [60], and biodegradable PHBV-HA (hydroxyapatite) composites had been employed for bone tissue engineering [59,61]. Sultana et al. reported that PHBV-based scaffolds fabricated via an emulsion freezing/freeze-drying technique were favorable sites for osteoblastic cells and are promising for application in bone tissue engineering [62]. Nana Wang et al. also demonstrated the potential application of β-Ca2SiO4/PHBV composites in bone tissue engineering. In this study, they found that β-Ca2SiO4/PHBV composite scaffolds could facilitate the adhesion and proliferation of human osteoblast-like MG-63 cells by stimulating the transcription of the transforming growth factor-β1 (TGF-β1) and bone morphogenetic protein-7 (BMP-7) genes. These scaffolds also induce early differentiation by promoting the transcription of ALP [63].

#### 7.1.4. PLLA (poly-l-lactic acid)

**Figure 9.**
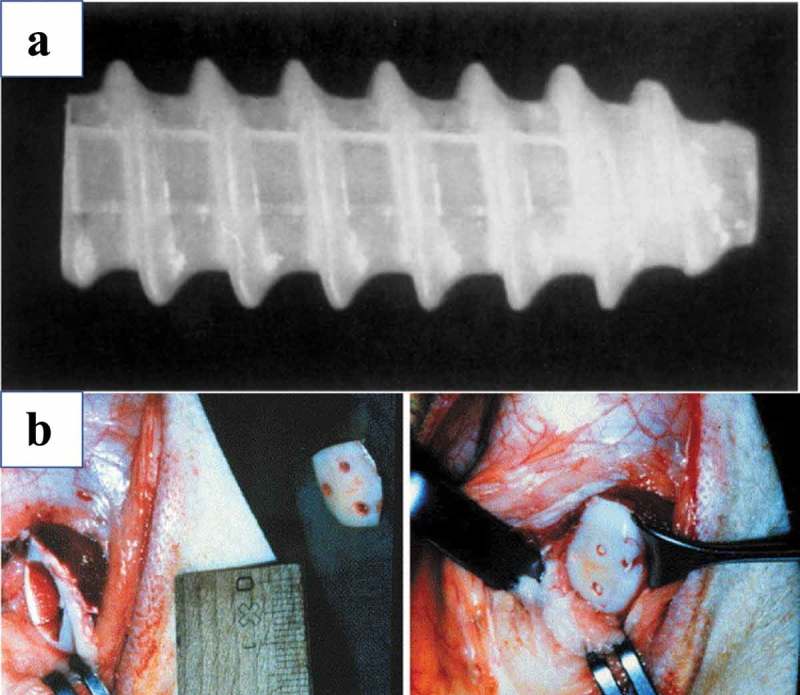
(a) Bioscrew composed of poly L-lactic acid [68]. Copyright 1995, Elsevier. (b) Osteotomy and osteotomy fixation using three poly-L/DL-lactide pins [70]. Copyright, 2004, Elsevier.

Semi-crystalline PLLA can generate piezoelectricity without poling, and this means the mechanical orientation of the molecules in the crystals and the quasi-crystalline region could directly respond to mechanical deformation to produce electricity. This occurrence has been attributed to the displacement of the C = O bond in PLLA in response to mechanical stress leading to the generation of a net dipole moment and charge [64,65]. The piezoelectric coefficient of PLLA (d_14_) is −10 pC/N [66]. Fukada and colleagues were one of the first teams to use PLLA as bone substitutes and have demonstrated that implantation of PLLA can promote bone growth in response to its piezoelectric polarization [67]. In this prior study, fracture healing and callus formation of cat tibiae were promoted as the draw ratio of the PLLA rod was increased. These results revealed that currents could be transformed from mechanical strain, while movement in piezoelectric PLLA scaffolds can help promote bone growth. Due to its biodegradability, non-toxicity, and advantageous mechanical properties, PLLA has substantial promise for use in clinical applications as biodegradable screws, fixation pins, and suture anchors in an effort to avoid a second surgery for removal of the implants [68–70] (Figure 9). These types of absorbable screws and pins have been gaining in their clinical use, particularly in cases where high mechanical stiffness and/or strength is not required [71].

### 7.2. Piezoceramics

In general, cytotoxicity is the major problem that limits the application of piezoceramics in the field of tissue engineering, especially those involving lead-based systems [72]. However, most of piezoceramics are available with a very high piezoelectric coefficient. Lead-free piezoceramics, which display a somewhat dose-dependent toxicity, such as barium titanate (BT), hydroxyapatite (HA), boron nitride (BNNT), and zinc oxide (ZO), can provide a better alternative choice to lead-based systems [47,73–75].

#### 7.2.1. Barium titanate

**Figure 10.**
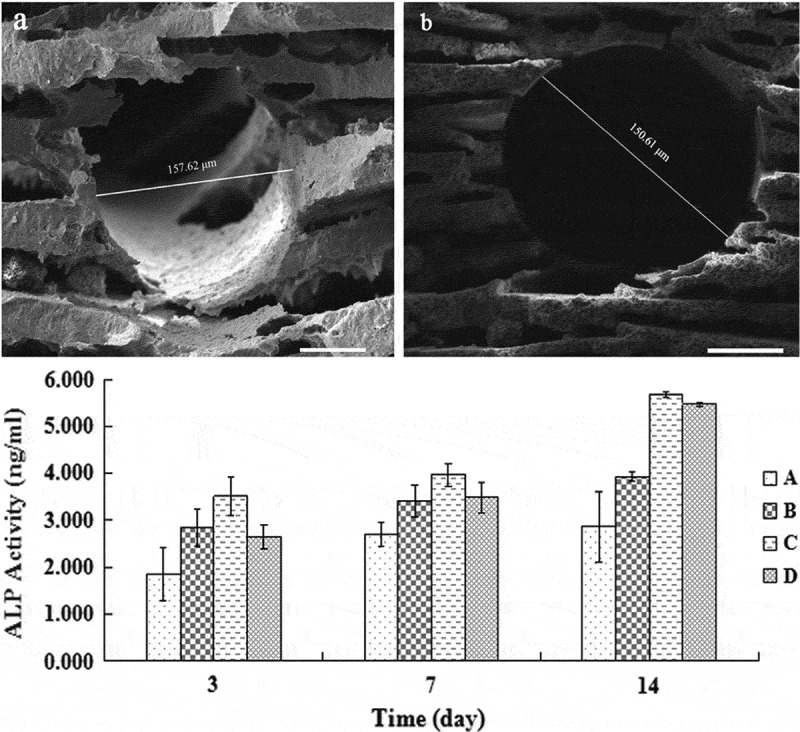
The porous structure of HA/BaTiO3 piezoelectric composites in SEM images. MG63 cells demonstrated better ALP activity in porous HA/BaTiO3 composites. Dense HA/BaTiO3 and porous HA/BaTiO3 with porosities of 40%, 50%, and 60% were set as group A, B, C, and D, respectively [81]. Copyright, 2015, Elsevier B.V.

Barium titanate (BT) is an inorganic compound with the chemical formula BaTiO3. This compound is highly biocompatible and cytocompatible with a piezoelectric coefficient (d_33_) of 191 pC/N [76,77]. Barium titanate nanoparticles have been shown to enhance osteogenic differentiation of mesenchymal stem cells [78,79], and these results provide for new approaches in bone tissue engineering. Furthermore, Ehterami et al. reported that highly porous BT scaffolds coated with Gel/HA nanocomposites presented with good biocompatibility, and MG63 osteoblast-like cells exhibited better adhesion, proliferation, and migration into the pores of these scaffolds [80]. In recent years, HA/BT (hydroxyapatite/barium titanate) composites have gathered significant interest within the scientific community for use in the design of scaffolds for bone substitutes owing to their bioactivity and osteogenic capabilities [81–84] (Figure 10).

#### 7.2.2. HA (Hydroxyapatite)

HA is a naturally occurring mineral form of calcium apatite and is present in the bones and teeth of human beings, and HA is an essential element required for bone regeneration. The piezoelectric coefficient of HA is in the range of 1.5 to 2.4 pC/N [85]. This bioceramic has been widely used as an artificial bone substitute because of its favorable biological properties, including high biocompatibility, bioaffinity, bioactivity, osteoconduction, and osteointegration [86,87]. In addition, HA has also been used in composite piezoelectric materials in the field of bone repair and tissue engineering. The introduction of a piezoelectric phase can promote the adhesion and proliferation of mouse fibroblast and human osteoblast [88], and scaffolds with porous structures made using polarized HA or HA composite materials can enhance the cellular response during bone regeneration. Kumar et al. demonstrated that cell attachment, proliferation, and metabolic activities were significantly increased when treated with HA porous scaffolds having a charged surface [89]. Aligned porous barium titanate/hydroxyapatite composites made by Yan Zhang et al. demonstrated high piezoelectric coefficients for use in bone tissue engineering without cytotoxicity [82].

#### 7.2.3. Boron nitride

**Figure 11.**
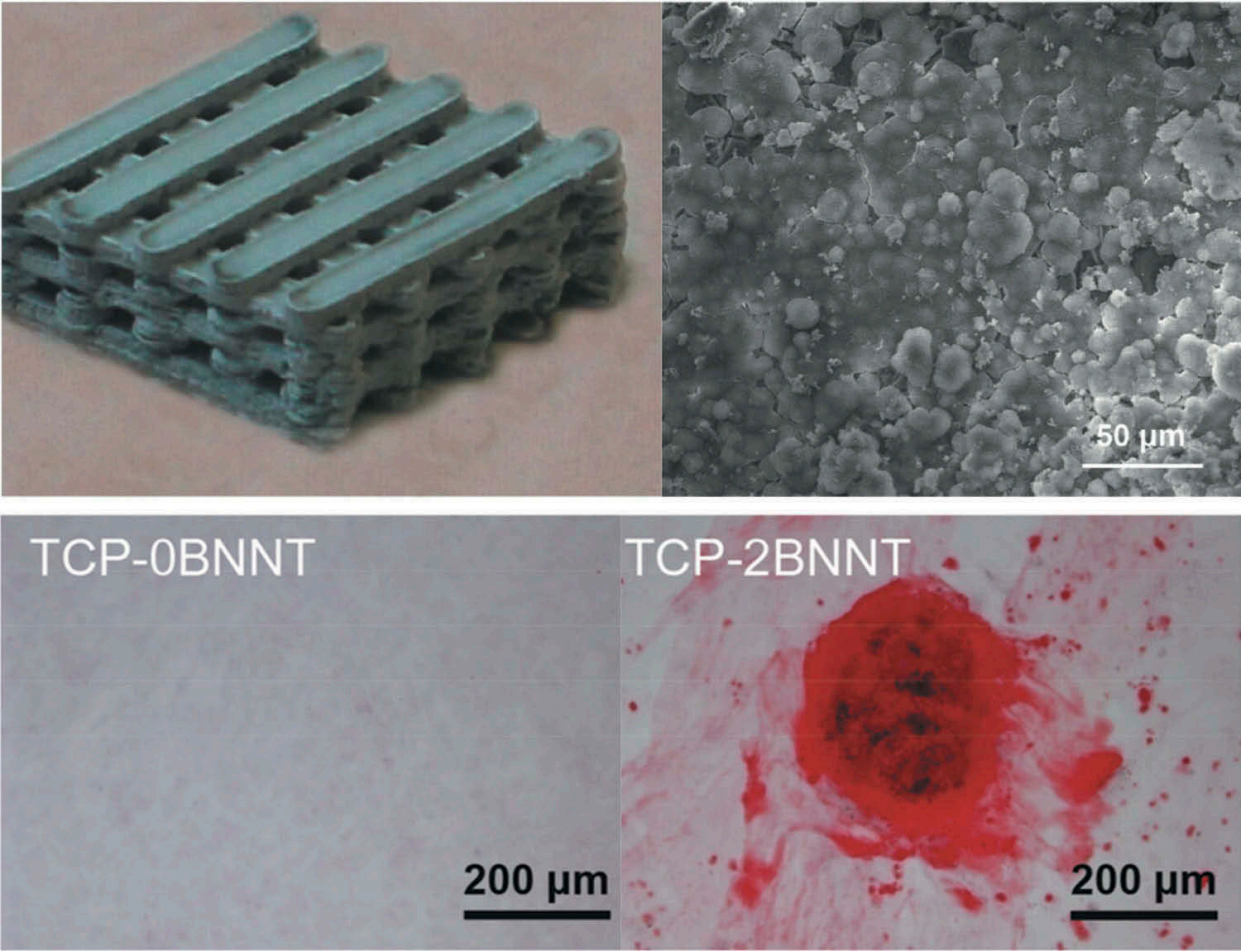
BNNT/TCP bone scaffold and SEM image (upper). The increase in BNNT content resulted in the formation of more calcium deposits (below) [93]. Copyright, 2016, American Scientific Publishers.

Boron nitride nanotubes (BNNTs) have shown great potential for practical use in many areas due to their excellent intrinsic properties, including superior mechanical strength, high thermal conductivity, electrically insulating behavior, piezoelectric property, neutron shielding capability, and oxidation resistance [90]. The piezoelectric property of BNNT is superior to that of piezoelectric polymers, with a d_33_ coefficient of 0.3 pC/N [91]. Over the last few years, BNNT has gained increasing attention in the field of osteogenesis, owing to its favorable biocompatibility, large specific surface area, and superior mechanical properties [92]. BNNT composite scaffolds have been shown to have a positive influence on osteogenesis and osteoinductive properties, owing to more calcium deposition and up-regulated expression levels of osteoblast markers, as presented in the study by Shuai et al [93]. (Figure 11). Xia Li et al. also presented similar results indicating that a BNNT layer could promote the attachment and growth of mesenchymal stem cells and enhance ALP activity, a marker of osteogenic differentiation [94]. Moreover, the positive influence on cell proliferation and attachment to BNNT seems to be exclusively related to osteoblasts, but not on chondrocytes, fibroblasts, or smooth muscle cells [95]. Therefore, BNNT is potentially useful for bone regeneration and orthopedic applications.

#### 7.2.4. ZnO (zinc oxide)

**Figure 12.**
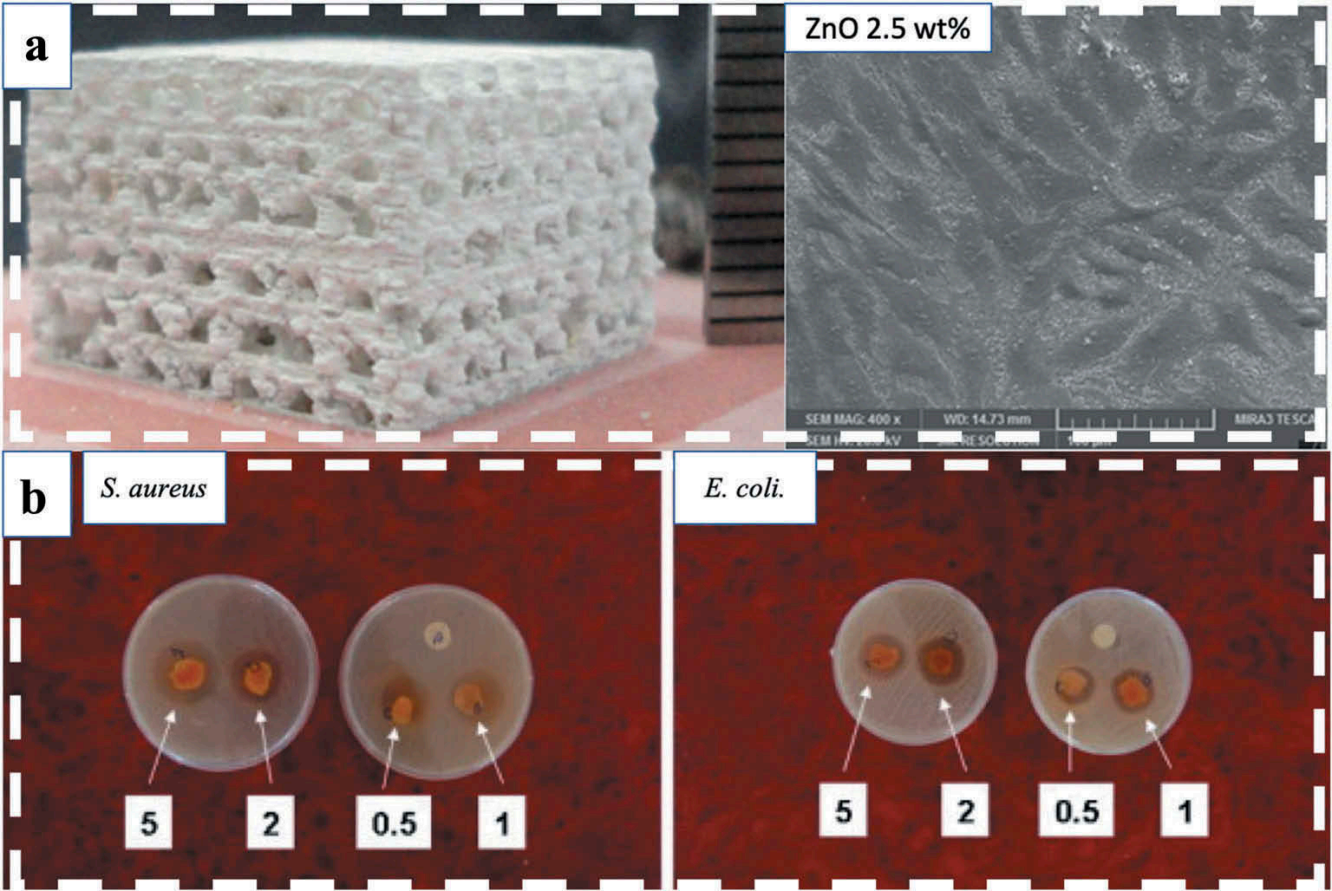
(a) In the ZnO/β-TCP composite interconnected porous scaffold, fracture toughness, compressive strength, cell attachment, and cell proliferation improve at optimal ZnO contents [101]. (b) Antibacterial activity of sodium alginate (SA)/poly (vinyl alcohol) (PVA)/ZnO fibers towards *S. aureus* (left) and *E. coli* (right) at various concentrations (0.5, 1, 2, and 5 wt%) of ZnO nanoparticles [102]. Copyright, 2011, Elsevier B.V.

ZnO has seen a large number of applications in the fields of environmental science, biomedicine, and electronics because of its high biocompatibility and ideal antimicrobial, physical, and chemical properties [96]. The piezoelectric coefficient of ZnO is 12.4 pC/N, and it has promising piezoelectric properties, promoting its use as nanogenerators for tissue engineering [97–99]. Without any external physical stimulus, the piezoelectric potential induced by mechanical force from the movement of living cells on an underlying ZnO nanosheet array can achieve the opening of voltage-gated calcium channels (VGCCs) or stretch-activated cation channels (SACCs). Subsequently, the opening of calcium channels within the plasma membrane of osteoblast-like cells results in high amplitudes of Ca^2+^ transients and the modulation of cell motility and activity [100]. In recent years, ZnO has also been used for the improvement of the mechanical and degradation properties of some bio-glasses, such as hydroxyapatite and tricalcium phosphate, which are known for their poor mechanical properties and fast degradation but high biocompatibility. The incorporation of ZnO nanoparticles largely increases the fracture toughness and compressive strength of bioceramics, and it also increases the bioactivity of these composites. Improved attachment and cell proliferation of osteoblast-like human cells were observed on the surface of composite scaffolds when the content of ZnO was increased from 0 to 2.5 wt% [101] (Figure 12(a)). In addition, the antibacterial properties of ZnO also help increase the application of ZnO-based composites in many biomedical fields [102] (Figure 12(b)).

## 8. Conclusions

Bone is a complex organ possessing both physicomechanical and bioelectrochemical properties. The present review details how osteocytes convert external mechanical stimuli into internal bioelectrical signals and the induction of intercellular cytokines. Piezoelectricity is just one of several mechanical responses of the bone matrix that allows osteocytes, osteoblasts, osteoclasts, and osteoprogenitors to react to changes in their environment. The alternating cell membrane potential of osteocytes and bone marrow stromal cells can initiate osteogenesis or osteoclastogenesis, and this is dependent on hyperpolarization or depolarization. Piezoelectric and triboelectric materials could be used as self-powered electrical generators to promote osteogenic proliferation and differentiation due to their electromechanical properties, which could promote the development of promising applications in tissue engineering and bone regeneration.
